# Structural tuning of organoruthenium compounds allows oxidative switch to control ER stress pathways and bypass multidrug resistance†

**DOI:** 10.1039/c6sc00268d

**Published:** 2016-03-01

**Authors:** Mun Juinn Chow, Cynthia Licona, Giorgia Pastorin, Georg Mellitzer, Wee Han Ang, Christian Gaiddon

**Affiliations:** a Department of Chemistry, National University of Singapore 3 Science Drive 3 117543 Singapore chmawh@nus.edu.sg +65 6516 5131; b NUS Graduate School for Integrative Sciences and Engineering Singapore; c U1113 INSERM 3 Avenue Molière Strasbourg 67200 France gaiddon@unistra.fr +33 68 52 53 56; d Section Oncology, FMTS, Strasbourg University Strasbourg France; e Department of Pharmacy, National University of Singapore 18 Science Drive 4 117543 Singapore

## Abstract

Multidrug resistance (MDR) is a major impediment to the success of chemotherapy in many cancer types. One particular MDR mechanism is the inherent or acquired adaptation of the cellular survival pathways that render malignant cells resistant to apoptotic cell death. Since most drugs act through apoptosis, compounds capable of inducing alternative forms of programmed cell death (PCD) can potentially be harnessed to bypass MDR. We investigated two organoruthenium complexes, RAS-1H and RAS-1T, and demonstrated that although they both induced non-apoptotic PCD through ER stress pathways, their modes-of-action were drastically different despite modest structural variations. RAS-1T acted through ROS-mediated ER stress while RAS-1H was ROS-independent. We further showed that they were more efficacious against apoptosis-resistant cells compared to clinical drugs including oxaliplatin. This work provides the basis for underpinning ER stress modulation using metal complexes to bypass apoptosis resistance.

## Abstract 2

Varying the arene ligand on organoruthenium compounds induced the differential activation of ER stress pathways, leading to non-apoptotic programmed cell death and bypassing drug resistance mechanisms.

## Graphical abstract

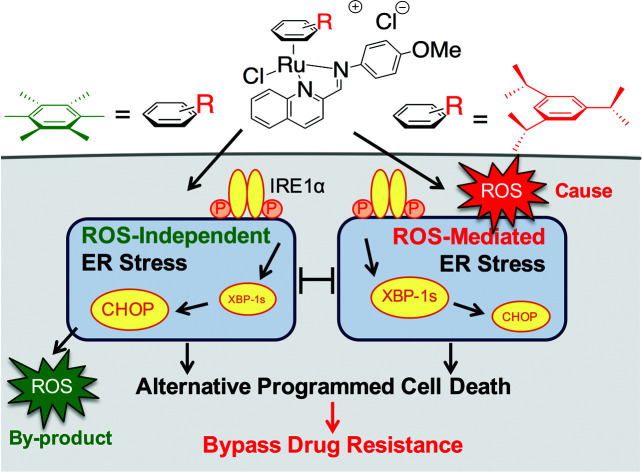

## Introduction

Chemotherapy remains one of the major treatment options for many cancer types.^1^ However, the effectiveness of chemotherapeutic treatments is frequently diminished^2^ due to the multidrug-resistance (MDR) phenotype found in many cancers that are associated with a poor clinical outcome such as gastric cancer, the third and fifth leading cause of cancer mortality in men and women worldwide, respectively.^3–6^ One dominant MDR mechanism in these cancers was determined to be the defective or selective adaptation of apoptotic pathways, which results in resistance to apoptosis.^7–11^ In addition, the overexpression of the membrane-bound ‘efflux’ transporter P-gp, one of the main causes of MDR, is also known to inhibit apoptosis by preventing caspase activation.^12–14^ Given that most clinically used drugs act by inducing apoptosis as their primary mode-of-action,^15,16^ it is not surprising that the success rates of chemotherapy in these cancers have been poor. One of the few strategies to overcome this mechanism of MDR involves the restoration of the expression or function of the pro-apoptotic gene in cancer cells through chemical or genetic modulation.^17,18^ A more attractive strategy would involve directly bypassing this mechanism of MDR entirely to induce cancer cell death *via* non-apoptotic programmed cell death (PCD).^19^

With the discovery of the antitumoural activity of cisplatin (CDDP), a widely used Pt^II^-based clinical drug for cancer chemotherapy, metallodrugs have received revived interest and attention in recent years.^20^ Yet, as with most clinical drugs including CDDP, the majority of the metallodrugs investigated act *via* apoptotic pathways and are therefore subject to classical MDR limitations. There have only been a handful of metallocomplexes shown to exert cytotoxicity *via* other alternative forms of PCD. Several Au-, Ru- and Fe-based complexes have exhibited the ability to induce type II autophagic cell death.^21–23^ A class of Cu^II^ thioxotriazole complexes and a Cu^I^ triazole–phosphine complex have demonstrated the ability to induce paraptosis in several cancer cell lines.^24,25^ More recently, two Re^V^ oxo complexes were reported to induce cell death *via* a form of programmed necrosis called necroptosis.^26^ However, their ability to overcome MDR mechanisms has never been validated. Therefore, new metallocomplexes with well-delineated modes of action, particularly *via* alternative PCD, could constitute a new strategy to overcome MDR.

**Fig. 1:**
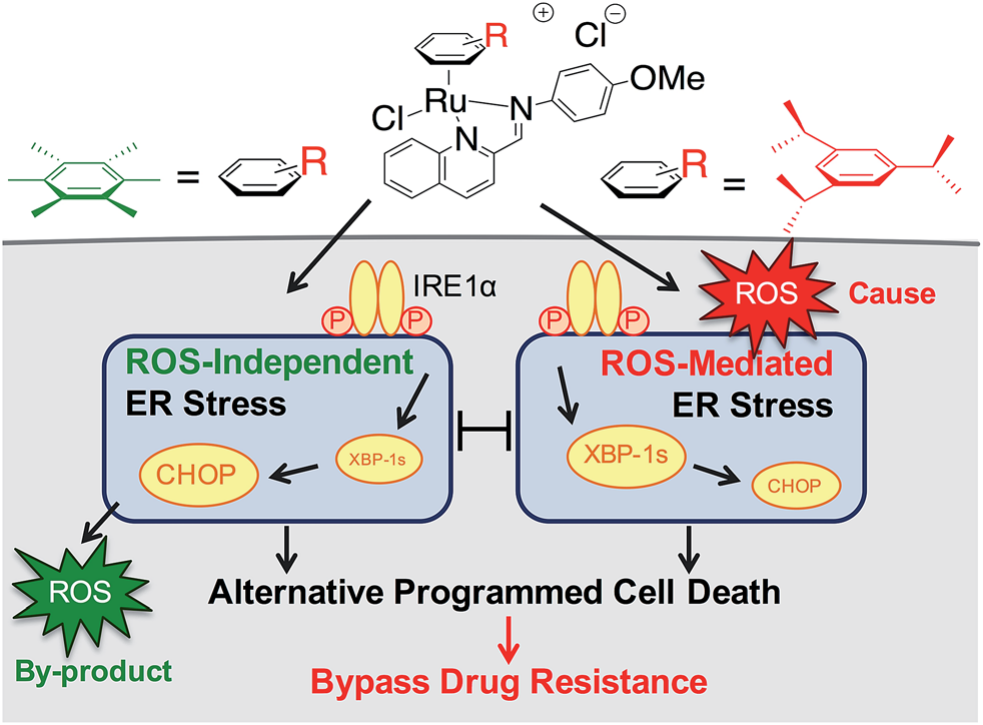
Differential ER stress pathway activation by RAS complexes leads to an alternative (non-apoptotic) PCD that bypasses drug resistance mechanisms.

We earlier reported the combinatorial synthesis and evaluation of a new class of water-soluble/stable half-sandwich Ru^II^ arene Schiff-base (RAS) complexes *via* a coordination-directed 3-component assembly.^27^ RAS-1T, which bore triisopropylbenzene (TIPB) and iminoquinoline ligands, was identified as a lead candidate as it was highly efficacious against several cancer cell lines yet exhibited attributes distinct from classical alkylating agents, such as CDDP and reported anticancer Ru^II^ complexes. For instance, RAS-1T was stable against hydrolysis and did not interact directly with dGMP nucleotides. In addition, it did not induce p53 expression in treated cells, commonly associated with DNA damage. Using gastric cancer as a model in this present study and in comparison with a newly synthesized hexamethylbenzene (HMB) analogue, RAS-1H, we demonstrate for the first time that varying the facially-bound arene ligands can have drastic effects on the compounds' mode-of-action leading to cell death, activating either ROS-dependent or ROS-independent ER stress pathways (Fig. 1). We show that the independent activation of both pathways leads to non-apoptotic PCD in treated cells and that this strategy could be harnessed to overcome apoptosis-resistance.

## Results and discussion

### Synthesis and characterization

**Scheme 1:**
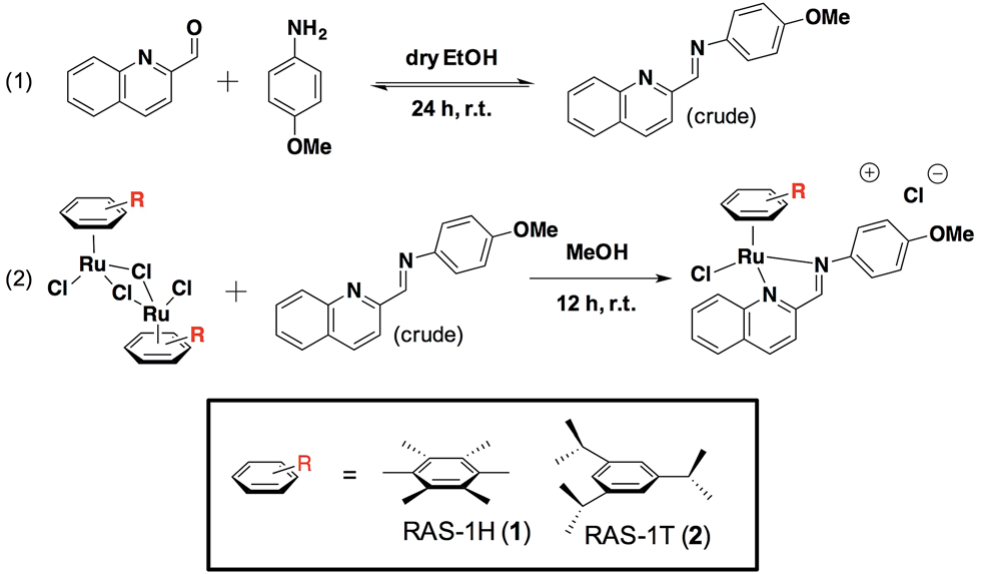
Synthesis route for the preparation of Ruthenium(ii) Schiff-base (RAS) complexes.

We previously reported the combinatorial synthesis of 450 distinct RAS complexes *via* the co-ordination of *in situ* assembled Schiff base ligands to organoruthenium scaffolds.^27^ Screening of this RAS library revealed RAS-1T to exhibit p53-independent activity and to be a lead candidate for a metallocomplex with antiproliferative activity that is distinct from classical alkylating agents, *e.g.* cisplatin, for further mode-of-action elucidation. We postulated that minor structural variations could influence cellular pathway activation, regardless of the similarities in their physicochemical properties. In order to discern how the structural variation of the arene ligand could influence the mode-of-action, we included the HMB-analogue RAS-1H in the present study. Both RAS-1H and RAS-1T were synthesized in good yields and purity (Scheme 1). The chelate ligand was prepared directly from 4-methoxylaniline and 2-formylquinoline *via* imine condensation and was treated with the desired [Ru(arene)Cl_2_]_2_ precursor (0.5 equiv., arene = HMB or TIPB) in MeOH. Purification *via* flash column chromatography gave both compounds in good yields. The complexes were characterized using ^1^H NMR and ESI-MS and their purity was confirmed using RP-HPLC and elemental analysis.

We compared the physicochemical properties of both complexes. Stability studies using UV-Vis spectroscopy showed that both complexes were stable to aquation, and were not prone to react with sulfur- or nitrogen-containing biomolecules (Fig. S4†). The lipophilicity of RAS-1H and RAS-1T was determined from their extent of partitioning between *n*-octanol and water. The log *P*_OW_ for RAS-1H and RAS-1T was determined to be −1.40 and −0.85, respectively, showing that both compounds were relatively hydrophilic despite having highly hydrophobic ligands, presumably due to the cationic nature of the compounds.

### Distinct cytotoxicity profiles of RAS-1H and RAS-1T

**Fig. 2:**
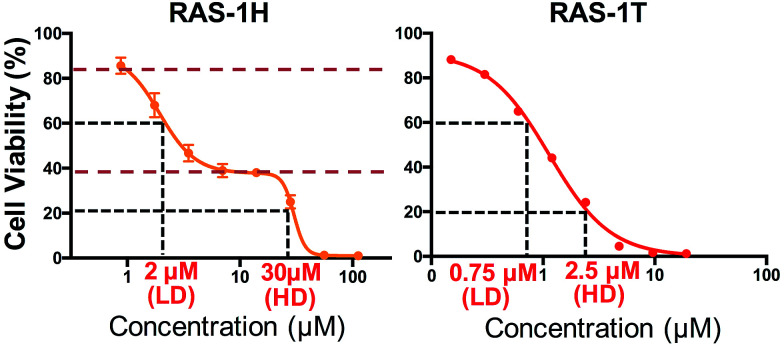
Varying the arene ligand on RAS complexes changes their antiproliferative profiles. Cell viability curves of compounds RAS-1H and RAS-1T. The curve represents the mean ± s.e.m. of three independent experiments. RAS-1H displayed a biphasic cytotoxicity curve unlike RAS-1T, hinting at a different mode-of-action. Two equipotent concentrations at low-dose (LD) and high-dose (HD) were chosen for cell treatment in proteins and mRNA expression studies.

We investigated the potentially contrasting mode-of-action of RAS-1H and RAS-1T by first testing their efficacy against a panel of gastric and colorectal cancer cell lines (Table S1†). As expected, RAS-1T displayed a low micromolar IC_50_ value in all 4 cell lines tested. The IC_50_ of RAS-1T was also 34 times and 7 times lower than that of cisplatin in the gastric cancer cell lines AGS and KATOIII, respectively. In comparison, RAS-1H demonstrated slightly more modest activity with IC_50_ values 11 times lower than cisplatin in AGS and 1.5 times higher in KATOIII. Closer scrutiny of the cytotoxicity curve of RAS-1H in AGS revealed a bi-phasic profile that was different from that of RAS-1T (Fig. 2). This suggested different modes-of-action for RAS-1H and RAS-1T. We determined two equipotent concentrations at low dose (LD) and high dose (HD) for each complex for cell treatment to further determine the difference in their mode-of-action.

### RAS-1H and RAS-1T induced early time-point ROS and activated the antioxidant defense mechanism

**Fig. 3:**
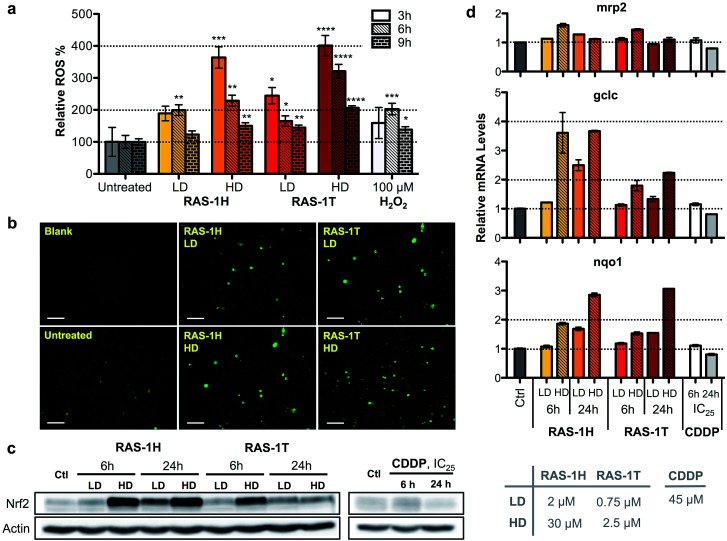
Complexes RAS-1H and RAS-1T induce early time-point ROS and activate the cellular antioxidant defence mechanism. (a) Detection of ROS with carboxy-H_2_DCFDA (20 μM) after treatment with RAS-1H and RAS-1T for 3 h, 6 h and 9 h using a microplate assay. Mean ± s.e.m. (*p < 0.05, **p < 0.01, ***p < 0.001, ****p < 0.0001; Student's *t* test). (b) Detection of ROS with a fluorescence microscope after treatment for 6 h. (c) Western blot analysis of Nrf-2, a central protein in cellular antioxidant defence and (d) expression levels of the Nrf-2 target gene in AGS cells after treatment with RAS-1H, RAS-1T and cisplatin at LD and HD for 6 h and 24 h. Homogeneous protein loading determined with reference to actin and gene expression normalized against *tbp* levels.

To unravel the mechanism of action of RAS-1H and RAS-1T, given the complex interplay of various possible pathways, we examined oxidative stress levels in treated AGS gastric cancer cells and the subsequent cellular response. Reactive oxygen species (ROS) generation has been implicated in the mode-of-action of many Ru^II^ complexes given the low energy barrier of the Ru^II^/Ru^III^ redox states, although it is not always clear that ROS generation was the cause of the observed cytotoxicity.^28–30^ Cellular ROS levels were determined using a commercial cell-permeable ROS probe, carboxy-H_2_DCFDA. The ROS levels were markedly increased in AGS cells treated with both RAS complexes in a time- and concentration-dependent manner. In cells treated with both complexes, ROS induction peaked at an early time-point of 3 h at similar levels before decreasing as indicated from the quantitative fluorescence measurements (Fig. 3a) and fluorescence microscopy images (Fig. 3b and S5†).

To gain an insight into the impact of ROS on the cells at relevant time-points (6 h and 24 h post-treatment), we examined the cellular response by looking into the antioxidant defense mechanism (Scheme S1†). Central to the antioxidant defense is the transcription factor Nrf-2,^31^ which is responsible for the regulation of several downstream target genes such as *gclc*,^32^*mrp2*,^33^ and *nqo1*,^34^ each having various roles in the mediation of oxidative stress. When ROS levels are elevated, Nrf-2 is activated and its target genes expression increases. Increased expression of Nrf-2 was observed after 6 h of treatment at HD for both RAS-1H and RAS-1T (Fig. 3c). Similarly, mRNA levels for all three Nrf-2 target genes increased after 6 h of treatment at HD (Fig. 3d). This was consistent with the induction of ROS observed at early-time points. In contrast, cells treated with CDDP remained at basal levels. However, there were differences in the Nrf-2 expression and activity patterns for both compounds. Firstly, the protein levels for Nrf-2 were higher after RAS-1H treatment compared to RAS-1T. Secondly, Nrf-2 induction remained elevated after 24 h for both concentrations of RAS-1H while Nrf-2 returned to basal levels after 24 h for RAS-1T. Lastly, the RNA level of *gclc* was more significantly increased by RAS-1H than RAS-1T. Taken together, the results indicate that the antioxidant defense for RAS-1H was switched on for an extended duration, underscoring the mechanistic differences in oxidative stress induction between the RAS complexes despite similar early time-point ROS production.

### Differential induction of the ER stress pathway by RAS-1H and RAS-1T

The relationship between ROS and endoplasmic reticulum (ER) stress is well-established and ROS could occur either upstream (cause) or downstream (product) of ER stress.^35,36^ ER stress can be characterized by the unfolded protein response (UPR), which can lead to recovery, cellular dysfunction or cell death.^37^ Three distinct UPR signaling pathways have been identified, namely the PERK/eif2α, IRE1α/XPB-1s and ATF6 pathways (Scheme S2†).^38^ Since elevated ROS levels point towards ER stress and UPR, we investigated if RAS-1H and RAS-1T induced ER stress biomarkers and the implications to their antiproliferative activity. RAS-1H and RAS-1T induced ER stress *via* the IRE1α/XPB-1s pathway as seen from the increased accumulation of XBP-1s after 6 h of treatment at HD (Fig. S6†). The induction of the downstream target CHOP after both 6 h and 24 h of HD treatment also confirmed the ER stress induction.^39^ While RAS-1H induced high levels of CHOP expression, RAS-1T favored XBP-1s splicing, alluding to different signaling pathways.

**Fig. 4:**
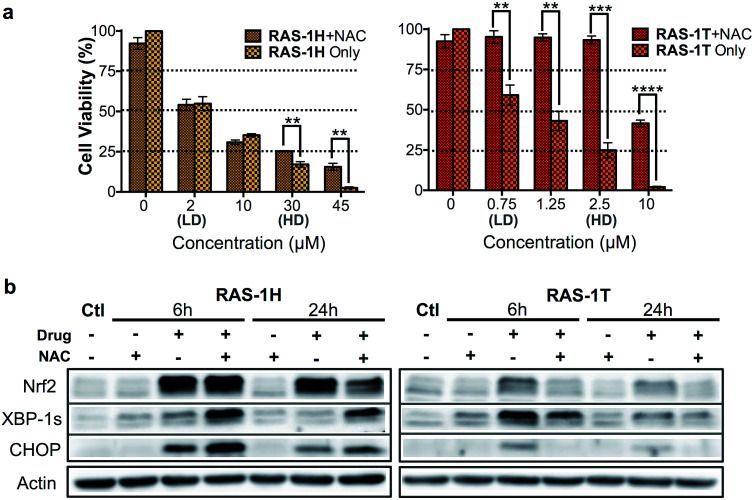
Differential activation of ROS-independent and ROS-mediated ER stress pathways by RAS-1H and RAS-1T. (a) Cell viability (%) of AGS cells treated with RAS-1H and RAS-1T for 48 h with and without NAC (2 mM). Mean ± s.e.m. (**p < 0.01, ***p < 0.001, ****p < 0.0001; two-tailed Student's *t*-test). (b) Western blot analysis of ER stress protein markers in AGS cells after treatment with RAS-1H and RAS-1T at HD, with and without NAC (2 mM). Homogeneous protein loading determined with reference to actin.

We therefore examined whether ROS production and the subsequent ER stress were critical to the antiproliferative activity of RAS-1H and RAS-1T on AGS cells. We employed *N*-acetylcysteine (NAC) as a ROS quencher and determined the cell viability and protein level expression of Nrf-2, XBP-1s and CHOP under various conditions, in the absence or presence of NAC. In the case of RAS-1T, cytotoxicity up to 2.5 μM (HD) was completely removed by the co-treatment with NAC. At higher levels of RAS-1T exposure, *i.e.* 10 μM which typically led to the 100% loss of viable cells, 50% cell viability was restored with NAC co-treatment (Fig. 4a). Furthermore, Nrf-2 levels were low in cells co-treated with NAC (Fig. 4b) indicating that the antioxidant defense was not switched on. In keeping with these observations, ER stress markers XBP-1s and CHOP were also suppressed in cells co-treated with NAC, suggesting a low UPR and reduced ER stress. Taken together, the results point toward ROS production being critical for the antiproliferative activity of RAS-1T (causal) and it exerting its mode-of-action *via* ROS-mediated ER stress. In contrast, ROS quenching did not suppress RAS-1H-induced cell death at treatments of less than 30 μM (HD) (Fig. 4a). The NAC treatment did not block RAS-1H-induced Nrf-2 protein levels after 6 h of treatment and had only a partial effect at the 24 h time-point. Moreover, ROS quenching also did not suppress XBP-1s or CHOP induction (Fig. 4b). In fact, the co-treatment of RAS-1H with NAC increased ER stress as indicated by the marked increase in XBP-1s and CHOP accumulation. This suggested that the cell death induced by RAS-1H was not dependent on the elevated ROS levels (Fig. 3a) and that ROS production was not critical for its mode-of-action (by-product).

### RAS-1H and RAS-1T induce non-apoptotic programmed cell death

**Fig. 5:**
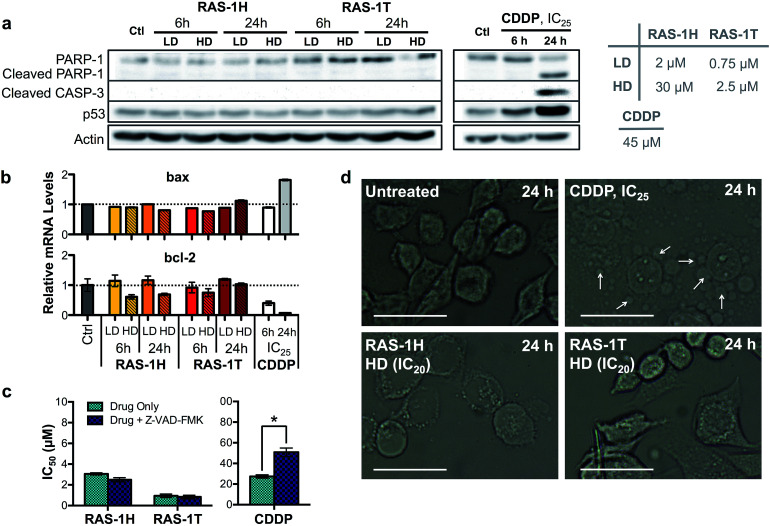
Complexes RAS-1H and RAS-1T induce non-apoptotic cell death. (a) Western blot analysis of proteins related to the apoptosis pathway and (b) expression levels of pro-apoptotic and anti-apoptotic genes in AGS cells after treatment with RAS-1H, RAS-1T and cisplatin at LD and HD for 6 h and 24 h. Homogeneous protein loading determined with reference to actin and gene expression normalized against *tbp* levels. (c) IC_50_ values of RAS-1H, RAS-1T and cisplatin after 48 h of treatment with and without an apoptosis inhibitor, Z-VAD-FMK (5 μM). Mean ± s.e.m. (*p < 0.05; two-tailed Student's *t*-test). (d) Microscope images of AGS cells treated with RAS-1H, RAS-1T and cisplatin for 24 h (scale bar = 100 μm). Examples of smaller apoptotic bodies due to ‘budding’ are pointed out with white arrows.

The contrasting yet well-defined antiproliferative pathways brought about by RAS-1H and RAS-1T, both of which invoked ROS production, could be harnessed to bypass the conventional apoptotic pathways induced by classical drugs such as CDDP. We therefore compared the apoptosis biomarkers commonly induced by CDDP treatment with the RAS complexes.^40,41^ Western blot analyses of AGS cells treated with CDDP revealed significant upregulation in p53 expression and cleavage of caspase 3 and PARP-1 (Fig. 5a). A concomitant increase of *bax* and decrease of *bcl-2* gene expression was also observed after 24 h (Fig. 5b), indicative of apoptosis induction. In contrast, cells treated with RAS-1H and RAS-1T did not exhibit such expression profiles, regardless of the concentration or duration of treatment. Furthermore, the two distinct hallmarks of apoptosis, namely (i) the activation of apoptosis-executor caspases, and (ii) morphological changes consistent with apoptosis were also absent in cells treated with RAS-1H and RAS-1T.^42^ The co-treatment of cells with a broad-spectrum caspase-inhibitor, Z-VAD-FMK, did not reduce the efficacy of either RAS-1H or RAS-1T, as seen from their unchanged IC_50_ values (Fig. 5c) but it provided cytoprotection from CDDP. In addition, the cell death morphology induced by RAS-1H and RAS-1T after 24 h was markedly different from that induced by CDDP (Fig. 5d and S7†). For cells treated with cisplatin, the ‘budding’ and formation of smaller apoptotic bodies were observed. In contrast, cells treated with RAS-1H and RAS-1T did not display the same morphological changes commonly seen in apoptotic cell death. To rule out the possibility of necrotic cell death, we also tested the activity of RAS-1H and RAS-1T in the absence and presence of IM-54, an inhibitor of oxidative stress-induced necrosis. The cell viability of cells treated with various concentrations of RAS-1H and RAS-1T did not change significantly in the presence of IM-54, indicating that necrosis was unlikely to be the mode of cell death (Fig. S8†). The lack of caspase involvement and the marked difference in cell death morphology compared to CDDP-treated cells suggested that RAS-1H and RAS-1T induced a form of non-apoptotic PCD.

### RAS-1H and RAS-1T bypass the apoptosis resistance mechanism in colorectal cancer cell lines

In order to validate the ability of the RAS complexes to bypass apoptosis resistance, we employed the apoptosis-resistant TC7 cell line as a functional cell model. TC7 is a cell line cloned from parental colorectal adenocarcinoma Caco-2 cells using limited dilution and is found to be highly resistant to conventional drug treatment.^43^ It has a p53-null status and a higher basal expression of anti-apoptotic Bcl-2 and Bcl-x_L_, as well as a lower expression of pro-apoptotic Bax. A previous study done on the activity of 5-fluorouracil on a panel of colorectal cancer cell lines including TC7 showed a positive correlation between their resistance to 5-fluorouracil treatment and their basal (Bcl-2 + Bcl-x_L_)/Bax expression ratio,^44^ suggesting that the drug-resistance observed in TC7 was due in part to apoptosis-resistance. The colorectal cancer cell lines HCT116 and HT-29 were used as non-resistant models. The activities of RAS-1H and RAS-1T were compared to the clinical drugs oxaliplatin (OXP), etoposide (ETOP), 5-fluorouracil (5-FU) and doxorubicin (DOX) using their resistance factor (RF), expressed as a factor of IC_50_ [TC7] against either IC_50_ [HT-29] or IC_50_ [HCT116]. OXP was used instead of CDDP as it is the leading drug for the treatment of colorectal cancer for which CDDP is poorly efficacious.

**Fig. 6:**
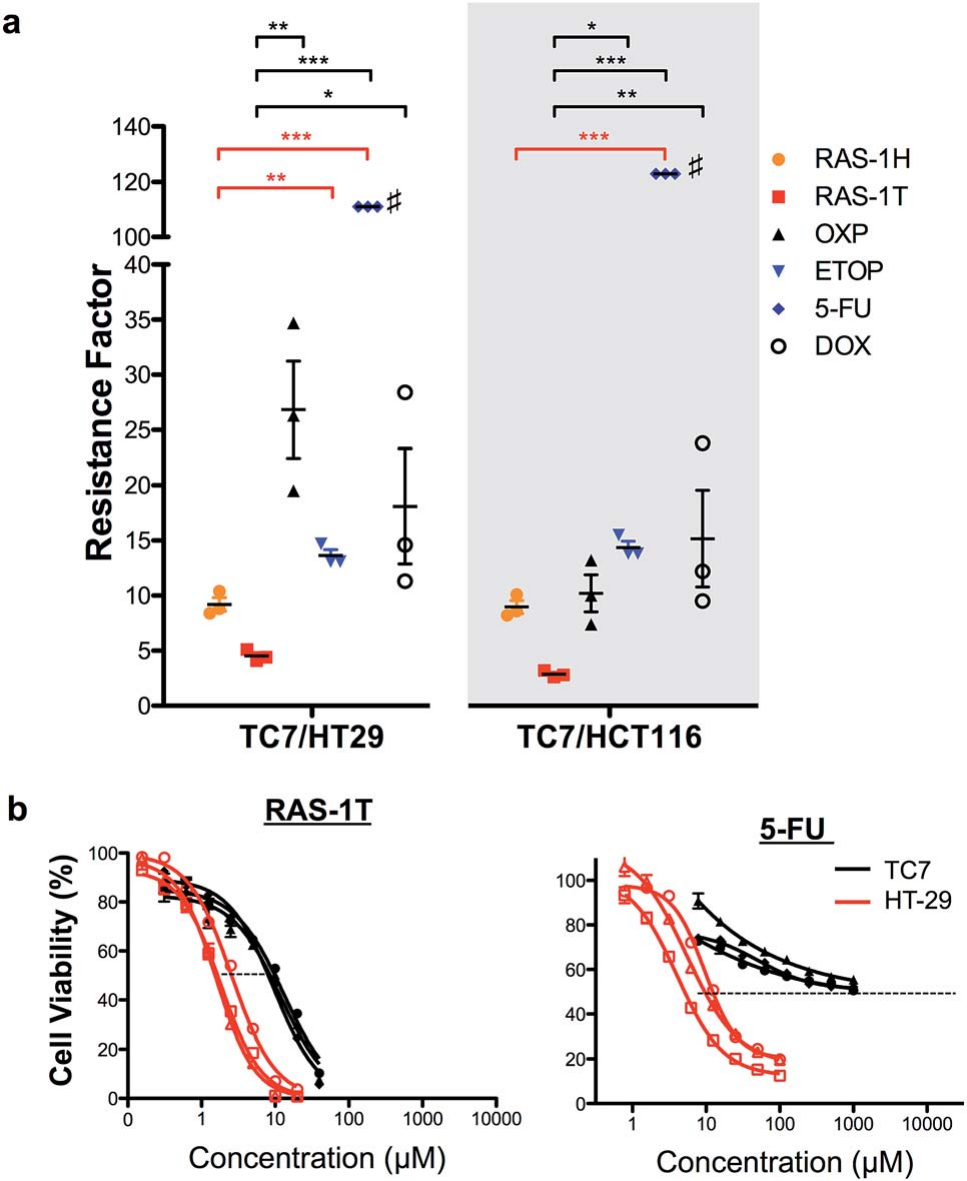
RAS-1H and RAS-1T are least affected by the resistance mechanism of drug-resistant TC7 cells, compared to other clinically approved drugs. (a) Resistance factors are calculated by taking the ratio of IC_50_ from 3 independent experiments in apoptosis-resistant TC7 over the mean IC_50_ in non-resistant HT29 and HCT116. Mean ± s.e.m. (*p < 0.05, **p < 0.01, ***p < 0.001; one-way ANOVA test with Tukey post-hoc test). ^#^IC_50_ in TC7 > 1000 μM in all 3 experiments and the resistance factor shown is the lowest possible. (b) Comparison of the cell viability curves for RAS-1T and 5-FU in TC7 and HT-29.

Both RAS-1H and RAS-1T were the least affected by TC7 compared to the frequently used clinical drugs (Fig. 6 and S9, and Table S2†). In particular, RAS-1T exhibited the lowest resistance factors (RFs) amongst all tested compounds of 4.5 (TC7/HT-29) and 2.8 (TC7/HCT116) while the RAS-1H values were 9.2 and 9.0, respectively. In contrast, the RF values for OXP were 26.9 and 10.2 while 5-FU exhibited RFs exceeding 111 and 123. Furthermore, RAS-1T also exhibited low micromolar IC_50_ values in TC7, highlighting its high efficacy in the apoptosis-resistant cell lineage. These results suggested that the apoptosis-resistance of TC7 did not significantly impact the effectiveness of both RAS complexes, unlike the tested clinical drugs, thereby validating in a functional cell model the hypothesis that RAS-1H and RAS-1T could bypass apoptosis resistance *via* the induction of alternative PCDs (Scheme S3†).

## Conclusion

The variation of the arene ligand in half-sandwich Ru complexes is commonly used as a means to modulate physical properties such as hydrophobicity and solubility. Limited studies have been done on how arene ligands could influence the cellular mode-of-action. Two separate studies on a class of Ru(ii)–arene complexes bearing ethylenediamine ligands have demonstrated that arene ligands, with varying hydrophobicity and π-acidity, could be used to modulate the rate of hydrolysis and extent of π-stacking of DNA bases.^45,46^ A separate study showed that the functionalization of the arene with a maleimide moiety allowed for the selective delivery of the complexes *via* selective binding with thiol-containing biomolecules.^47^ However, these studies focused on how arene modification could be used to modulate the physicochemical properties and target-binding ability without demonstrating any differential activation of cellular pathways.

In contrast, our current study represents one of the few such studies, demonstrating that a subtle change in the arene ligands on the RAS complexes had a drastic effect on its mode-of-action, switching the ability of the drug to induce cell death from a ROS-mediated ER stress pathway to a ROS-independent pathway. Since the variation of the arene ligand did not significantly change the physiochemical properties such as stability, reactivity to biomolecules or hydrophobicity, the observed difference is most likely influenced directly by the site-specific structural changes of the arene ligand. Although the induction of ER stress by other metal complexes has been previously reported,^30,48,49^ mechanistic insights into ER stress activation and the subsequent implications of structural factors were not discussed. Our current study provides the first molecular basis for ER stress activation, highlighting the complex relationship between the structure of the compound and its impact on the mode-of-action *via* ER stress. It is noteworthy that the structural tuning also impacted its ability to bypass cancer cell MDR mechanisms, as seen in the two-fold difference in the resistance factor between RAS-1H and RAS-1T. These factors should be taken into consideration when designing such compounds as anticancer agents to improve the treatment outcome for MDR cancers. Based on the current study, we hypothesized that the structural change to the RAS complexes could have affected the selectivity of binding to their target, especially if the cellular target contains several homologous isoforms. This could have caused the observed differences in the mode-of-action between the two complexes. Nevertheless, more studies are required to identify the cellular target(s) of RAS-1H and RAS-1T before this can be validated.

## Conflict of interest

The authors declare no competing financial interest.

## Supplementary Material

SC-007-C6SC00268D-s001
