# Correction: Structural tuning of organoruthenium compounds allows oxidative switch to control ER stress pathways and bypass multidrug resistance

**DOI:** 10.1039/d3sc90157b

**Published:** 2023-08-29

**Authors:** Mun Juinn Chow, Cynthia Licona, Giorgia Pastorin, Georg Mellitzer, Wee Han Ang, Christian Gaiddon

**Affiliations:** a Department of Chemistry, National University of Singapore 3 Science Drive 3 117543 Singapore chmawh@nus.edu.sg +65 6516 5131; b NUS Graduate School for Integrative Sciences and Engineering Singapore; c U1113 INSERM 3 Avenue Molière Strasbourg 67200 France gaiddon@unistra.fr +33 68 52 53 56; d Section Oncology, FMTS, Strasbourg University Strasbourg France; e Department of Pharmacy, National University of Singapore 18 Science Drive 4 117543 Singapore

## Abstract

Correction for ‘Structural tuning of organoruthenium compounds allows oxidative switch to control ER stress pathways and bypass multidrug resistance’ by Mun Juinn Chow *et al.*, *Chem. Sci.*, 2016, **7**, 4117–4124, https://doi.org/10.1039/C6SC00268D.

The authors regret that an incorrect version of **Fig. 3** was included in the original article, where two incorrect images were used, namely RAS-1H LD treatment and RAS-1H HD treatment, resulting in an unintentional duplication. The correct version of **Fig. 3** is presented here, which is now consistent with Fig. S5a from the ESI of the original publication.


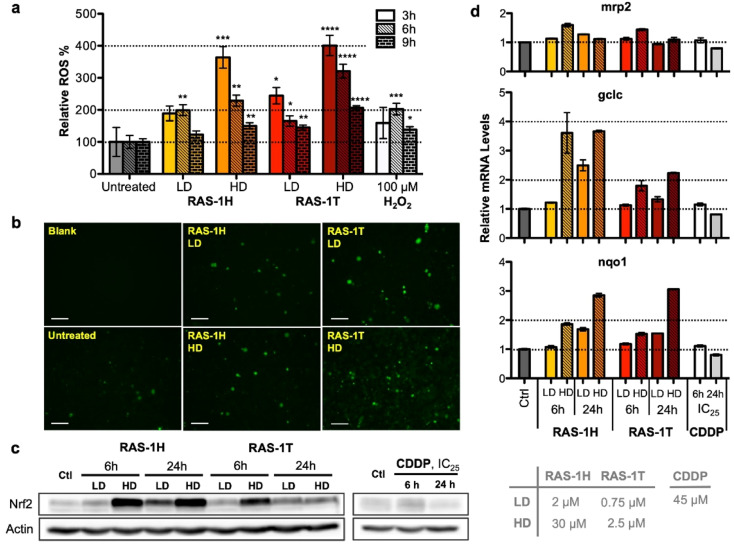
 **Fig. 3** Complexes RAS-1H and RAS-1T induce early time-point ROS and activate cellular antioxidant defense mechanism. (a) Detection of ROS with carboxy-H_2_DCFDA (20 μM) after treatment with RAS-1H and RAS-1T for 3 h, 6 h and 9 h using a microplate assay. Mean ± s.e.m. (*p < 0.05, **p < 0.01, ***p < 0.001, ****p < 0.0001; Student's *t* test). (b) Detection of ROS with a fluorescence microscope after treatment for 6 h. (c) Western blot analysis of Nrf-2, a central protein in cellular antioxidant defence and (d) expression levels of Nrf-2 target gene in AGS cells after treatment with RAS-1H, RAS-1T and cisplatin at LD and HD for 6 h and 24 h. Homogeneous protein loading determined with reference to actin and gene expression normalized against *tbp* levels.

The Royal Society of Chemistry apologises for these errors and any consequent inconvenience to authors and readers.

## Supplementary Material

