# Supplementary material for: Structural tuning of organoruthenium compounds allows oxidative switch to control ER stress pathways and bypass multidrug resistance
Source: Chem Sci. 2016 Mar 1;7(7):4117–24. doi: 10.1039/c6sc00268d (PMC6013925; doi:10.1039/c6sc00268d)
Supplement: SC-007-C6SC00268D-s001 [file SC-007-C6SC00268D-s001.pdf]

Electronic Supplementary Material (ESI) for Chemical Science.
This journal is © The Royal Society of Chemistry 2016

Supporting Information for

Structural tuning of organoruthenium compounds allows oxidative switch to control ER stress pathways and bypass multidrug resistance

Mun Juinn Chow[1,2], Cynthia Licona[4,5], Giorgia Pastorin[2,3], Georg Mellitzer[4,5], Wee Han Ang[,1,2] and Christian Gaiddon*[,4,5]*

[1]Department of Chemistry, National University of Singapore, 3 Science Drive 3, 117543 Singapore

[2]NUS Graduate School for Integrative Sciences and Engineering, Singapore

[3]Department of Pharmacy, National University of Singapore, 18 Science Drive 4, 117543 Singapore

[4]Université de Strasbourg, Strasbourg, France

[5]U1113 INSERM, 3 Avenue Molière, Strasbourg 67200, France

*To whom correspondence should be addressed:

Christian Gaiddon: gaiddon@unistra.fr

Wee Han Ang: chmawh@nus.edu.sg

EXPERIMENTAL

Materials. All experimental procedures were carried out without additional precautions to exclude air or moisture unless otherwise specified. All chemicals and solvents were used as received. $RuCl_3.xH_2O$ was purchased from both Precious Metals Online. $[(\eta^6$-hexamethylbenzene)$RuCl_2]_2$ and $[(\eta^6$-1,3,5-triisopropylbenzene)$RuCl_2]_2$ were synthesized according to previously reported protocols.[1] Thiazolyl blue tetrazolium bromide (MTT), Trizma® Base BioUltra, Nonidet P-40, DL-Dithiothreitol, Non-fat Dried Milk Bovine, TWEEN® 20, Ponceau S, N-acetylcysteine and IM-54 were purchased from Sigma-Aldrich. All other chemicals used were purchased from Sigma-Aldrich (Singapore). Ultrapure water used was purified by a Milli-Q UV purification system (Sartorius Stedim Biotech SA). Molecular Probes® 5-(and-6)-carboxy-2',7'-dichloro-dihydrofluorescein diacetate mixed isomer, Gibco® Versene solution, Gibco® Trypsin/EDTA solution, Gibco® MEM Non-Essential Amino Acids solution (NEAA), 10% SDS solution, Penicillin-Streptomycin (10 000 U/mL), Dulbecco's Phosphate-Buffered Saline (10x), TRIzol® Reagent and Applied Biosystem® High Capacity cDNA Reverse Transcription Kit were purchased from Life Technologies. Hyclone™ RPMI 1640, DMEM medium and Fetal bovine serum (FBS) was purchased from Thermo Fisher Scientific Inc. Bio-rad Protein Assay Dye Reagent Concentrate, 40% Acrylamide/Bis solution, 10x Tris/glycine buffer, TEMED, 4x Laemmli Sample Buffer, Nitrocellulose Membrane, 0.2 µm and 0.45 µm were purchased from Bio-rad Laboratories. cOmplete™, mini Protease Inhibitor Cocktail Tablets, RNAse A, FastStart Universal Probe Master (Rox) and FastStart Universal SYBR Green Master (Rox) were purchased from Roche Diagnostics. Luminata™ Classico and Luminata™ Crescendo Western HRP Substrate were purchased from Merck Millipore Corporation. Pan Caspase Inhibitor Z-VAD-FMK was purchased from R&D Systems™.

General instrumentation. ^{1}H NMR spectrums were obtained using a Bruker Avance 400 spectrometer and the chemical shifts (δ) were reported in parts per million with reference to residual solvent peaks. Electrospray-ionization Mass Spectrometry (ESI-MS) spectra were obtained using Thermo Finnigan MAT ESI-MS System. UV-vis spectra were obtained using the Shimadzu UV-1800 UV Spectrophotometer. Ru concentrations were determined using the Optima ICP-OES (Perkin-Elmer) operated by CMMAC, NUS. Elemental analyses of Ru complexes were carried out using a Perkin-Elmer PE 2400 elemental analyzer by CMMAC, NUS. Absorbance and fluorescence on 96-well plates were measured using TriStar^2 Multimode Reader LB942 from Berthold Technologies. mRNA was quantified using Thermo Scientific Nanodrop 1000 Spectrophotometer. Reverse Transcriptase-PCR was done using Applied Biosystems® 2720 Thermal cycler. qPCR was done using Applied Biosystems® 7500 Real Time PCR System. Fluorescence microscope images were taken with AxiomZoom.V16.

HPLC analysis of compound purity. Determination of the purity of RAS-1H was done using analytical HPLC on a Shimadzu Prominence System equipped with a DGU-20A$_3$ Degasser, two LC-20AD Liquid Chromatography Pump, a SPD-20A UV/Vis Detector and a Shim Pack GVP-ODS 2.0 mm 18 column (5 µM, 120Å, 250 mm x 4.60 mm i.d.) with detection at both 214 nm and 254 nm. The gradient elution conditions were as follows: 20-80% solvent B over 30 min, where solvent A is 10 mM aqueous NH_4OAc pH 7.0 and solvent B is CH_3CN.

Synthesis of RAS-1H, [(*η*^{6}-hexamethylbenzene)RuCl(4-methoxy-*N*-(2-quinolinyl-methylene)-aniline)]Cl. 2-quinolinecarboxaldehyde (78.5 mg, 0.5 mmol) and *p*-anisidine (61.5 mg, 0.5 mmol) were added to minimal amount of dry EtOH (5 mL) and stirred at r.t.

for 24 h. The solvent was removed *in vacuo*. The resulting crude yellowish brown solid [4-methoxy-*N*-(2-quinolinylmethylene)-aniline] was used for the next step without any further purification. [(η^6-hexamethylbenzene)$RuCl_2$]$_2$ (167. mg, 0.25 mmol) was then added to the crude 4-methoxy-*N*-(2-quinolinylmethylene)-aniline (131 mg, approx. 0.50 mmol) and stirred in MeOH (15 mL) for 12 hrs. The solvent was removed *in* vacuo and the resulting crude product was purified by silica gel colomn chromatography (Gradient elution: 1:4 v/v EtOH/$CHCl_3$, R_f = 0.4; 1:1 v/v EtOH/$CHCl_3$, R_f = 0.7). The final product was then dried *in vacuo* for 1 h to give a deep red solid. Yield: 110 mg (37%). ^{1}H NMR (400 MHz, DMSO-d6): δ 8.99 (s, 1H), 8.83 (d, *J* = 8.3 Hz, 1H), 8.37 (d, *J* = 8.8 Hz, 1H), 8.28 (t, *J* = 8.3 Hz, 2H), 8.15 (t, *J* = 8.6 Hz, 1H), 7.96 (m, 3H), 7.22 (d, *J* = 9.0 Hz, 2H), 3.88 (s, 3H), 1.75 (s, 18H) ppm. ESI-MS (+ve mode): m/z = 561 $[M]^+$. Analysis (Calcd., found for $C_{29}H_{32}N_2Cl_2ORu.0.5H_2O$): C (57.52, 57.55), H (5.49, 5.23) N (4.63, 4.58). RP-HPLC (% Purity): 98.1% at 214 nm and 98.6% at 254 nm; t_r = 19.8 min.

Synthesis of RAS-1T, [(η^6-1,3,5-triisopropybenzene)RuCl(4-methoxy-*N*-(2-quinolinyl-methylene)-aniline)]Cl. Complex RAS-1T was synthesized according to reported protocol.[1]

Cell lines and tissue culture. The human colorectal carcinoma cells HCT116, human colorectal adenocarcinoma cells SW480 and HT-29, human gastric adenocarcinoma AGS, human gastric carcinoma KATOIII were acquired from ATCC® (Manassa,VA). TC7 cells were cloned from parental colorectal adenocarcinoma Caco-2 by the limited dilution technique.[2] HCT116, HT-29 and SW480 cells were cultured in DMEM medium containing 10% FBS and 1% Penicillin/Streptomycin. TC7 cells were cultured in DMEM medium containing 20% FBS, 1% Penicillin/Streptomycin and 1% NEAA. AGS cells were cultured in RPMI 1640 medium containing 10% FBS and 1% Penicillin/Streptomycin. KATOIII cells

were cultured in RPMI 1640 medium containing 20% FBS and 1% Penicillin/Streptomycin. All cell lines were grown at 37 °C in a humidified atmosphere of 95% air and 5% CO_2. Experiments were performed on cells within 20 passages.

Determination of Log P_{ow}. Log P_{ow} of RAS-1H an RAS-1T were determined using the shake flask method. RAS-1H and RAS-1T were predissolved in ddH_2O that was presaturated with *n*-octanol (for 24 h and left to stand until phase separation occurs). The UV-vis spectrums for each samples were obtained and the absorbances at the λ_{max} of each compound were determined. Equal volume of *n*-octanol was added to each sample solution and the heterogeneous mixtures shaked for 2 h before centrifuging at 4000 rpm for 1 min to achieve phase separation. The final absorbance of the aqueous phase at the λ_{max} of each compound were determined and their water-octanol partition coefficient were calculated.

Inhibition of cell viability assay. The anti-proliferation activities of RAS-1H and RAS-1T on exponentially growing cancer cells were determined using MTT assay. HCT116, HT-29, SW480, AGS, KATOIII were seeded at 10 000 cells per well (100 µL) while TC7 cells were seeded at 5000 cells per well in Cellstar® 96-well plates (Greiner Bio-One) and incubated for 24 h. Thereafter, cancer cells were exposed to drugs at different concentration in media for 48 h. The final concentration of DMSO in medium was < 1% (v/v) at which cell viability was not significantly inhibited. The medium was removed and replaced with MTT solution (100 µL, 0.5 mg/mL) in media and incubated for an additional 45 min. Subsequently, the medium was aspirated, and the purple formazan crystals dissolved in DMSO (100 µL). The absorbance due to the dissolved purple formazan was than obtained at 565 nm. Inhibition to cell viability was evaluated with reference to the

IC_{50} value, which is defined as the concentration needed for a 50% reduction of survival based on the survival curves. IC_{50} values were calculated from the dose - response curves (cell viability vs. drug concentration) obtained in repeated experiments and adjusted to actual [Ru] administered, which was determined using ICP-OES. The experiments were performed in 4 replicates for each drug concentration and were carried out at least three times independently. For cell viability assays involving inhibitors, *N*-acetylcysteine (2 mM) and IM-54 (10 µM) were added together with test compounds and incubated for 48 h. Z-VAD-FMK (5 µM) was added 1 h prior to the addition of the test compounds. Cell viability in the absence and presence of inhibitor was normalized against untreated control. Experiments were performed in 4 replicates and carried out at least two times independently.

Antibodies and western blot protocol. AGS cells were grown at 500 000 cells per well (2 mL) on Cellstar® 6-well plates (Greiner Bio-One) for 24 h before being treated with RAS-1H and RAS-1T at LD and HD for 6 h and 24 h. Cisplatin treatment at 45 µM (IC_{25}) was used as a positive control for several experiments. The cells were lysed with lysis buffer [100 µL, 1% NP40, 150 mM NaCl, 50 mM Tris-HCl (pH 8.0), protease inhibitor]. The cell lysate was transferred to separate 2 mL tubes and sonicated for 10 s. The samples were then centrifuged at 13000 rpm, 4˚C for 15 min. The liquid supernatant containing the proteins were collected and total protein content of each sample was quantified *via* Bradford's assay. 50 µg of proteins from each sample were reconstituted in loading buffer [5% DTT, 1x Protein Loading Dye] and heated at 95˚C for 5 min. The protein mixtures were resolved on either a 10% or 15% SDS-PAGE gel by electrophoresis and transferred to a nitrocellulose membrane. The proteins bands were visualized *via* enhanced chemiluminescence imaging (PXi, Syngene) after treatment with the primary

antibodies and the appropriate secondary antibodies. Equal loading of protein was confirmed by comparison with actin expression. The following antibodies were used: p53 (FL-393), SQSTM1/p62 (D-3), GADD153 (F-168), Nrf-2 (sc13032) from Santa Cruz Biotechnologies. Cleaved caspase-3 (Asp175) from Cell Signaling Technology. PARP-1 (ab137653), β-Actin (ab75186) and GAPDH (ab9483) from Abcam. LC3 (NB100-2220) from Novus Biologicals. XBP-1s (Clone: 143F) from BioLegend. ECL Anti-rabbit IgG (NA934V) and ECL Anti-mouse IgG (NA931) from GE Healthcare Life Sciences. All antibodies were used at 1:500 dilutions except for actin (1:10000), GADPH, anti-mouse and anti-rabbit (1:5000).

Primers and qPCR protocol. Treatment conditions for AGS cells were similar to the protocol in western blot. RNA was extracted using TRIzol® Reagent and reverse transcription was performed with 2 µg of the extracted RNA using Applied Biosystem® High Capacity cDNA Reverse Transcription Kit with an Applied Biosystem® 2720 Thermal Cycler. Quantitative PCR was done on the resulting cDNA using FastStart Universal Probe Master (Rox) or FastStart Universal SYBR Green Master (Rox) with Applied Biosystem® 7500 Real Time PCR System. The relative starting quantities of genes of interest were normalized against the housekeeping genes TBP and samples were done in duplicates. Where SYBR probes are used, the specificity of the amplification was controlled by a melting curve. The gene and Assay ID of TaqMan probes are as follows: BAX (Hs00180269_m1), Bcl-2 (Hs00608023_m1) and TBP (Hs00427620_m1). Primer sequence for SYBR Green probes are as follows: Mrp2, 5'-CTG GAT CAC CTC CAA CAG GT-3' (forward) and 5'-AGA AGA CAG TCA GGT TCC CAA C-3' (reverse). GCLC, 5'-ATG CCA TGG GAT TTG GAA T-3' (forward) and 5'-GAT CAT AAA GGT ATC TGG CCT CA-3'(reverse). NQO1, 5'-GCC CAG ATA TTG TGG CTG A-3' (forward) and 5'-ACC

ACT GCA GGG GGA ACT-3’ (reverse). TBP, 5’-CGC TGG AAC TCG TCT CAC TA-3’ (forward) and 5’-GCC CAT AGT GAT CTT TGC AGT-3’ (reverse).

ROS Detection. AGS cells were seeded at 20,000 cells per well (100 µL) in black clear-bottom Cellstar® 96-well plates (Greiner Bio-One) and incubated for 24 h. Thereafter, the cells were exposed to RAS-1H and RAS-1T at LD and HD. H_2O_2 (100 µM) was used as a positive control. Separate plates were used for each time point of 3 h, 6 h and 9 h. Cells were washed once with HBSS and incubated with ROS probe, carboxy-H_2DCFDA (20 µM) in HBSS for 45 mins. The probe solution was then removed and replaced with 50 µL of HBSS. The fluorescence was measured using a microplate reader (excitation 485 nm/emission 535 nm). Experiments were performed in 8 replicates and carried out three times independently. For fluorescence images, cells were seeded in Cellstar® 24-well plates (Greiner Bio-One) at 200,000 cells per well. Treatment conditions were similar to the microplate measurements.

FIGURES

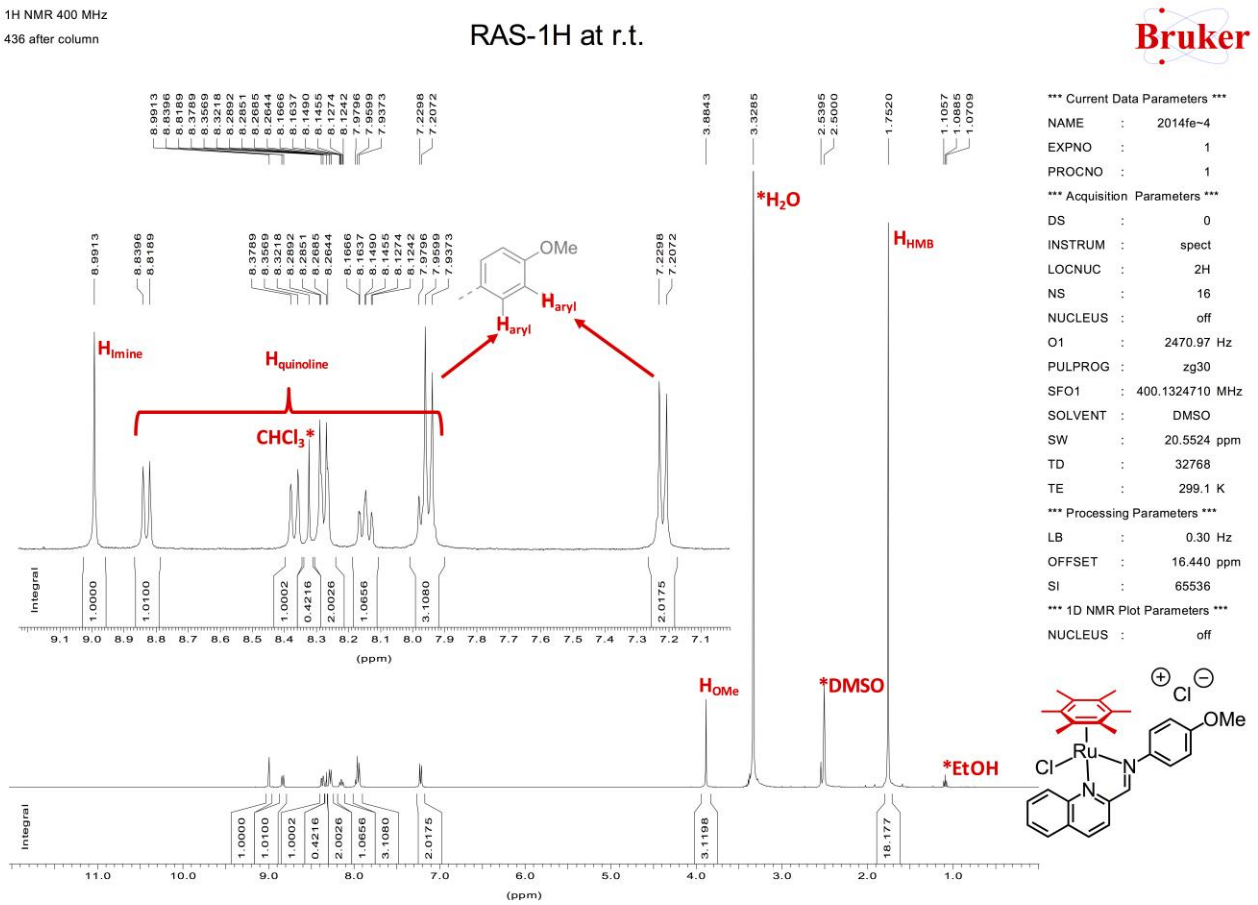

Figure S1. ^{1}H NMR Spectrum of RAS-1H. Spectrum was acquired using a 400 MHz spectrometer in DMSO-d6. The formation of the imine peak at 9.0 ppm is indicative of the formation of the complex. Residual solvent peaks are as indicated.

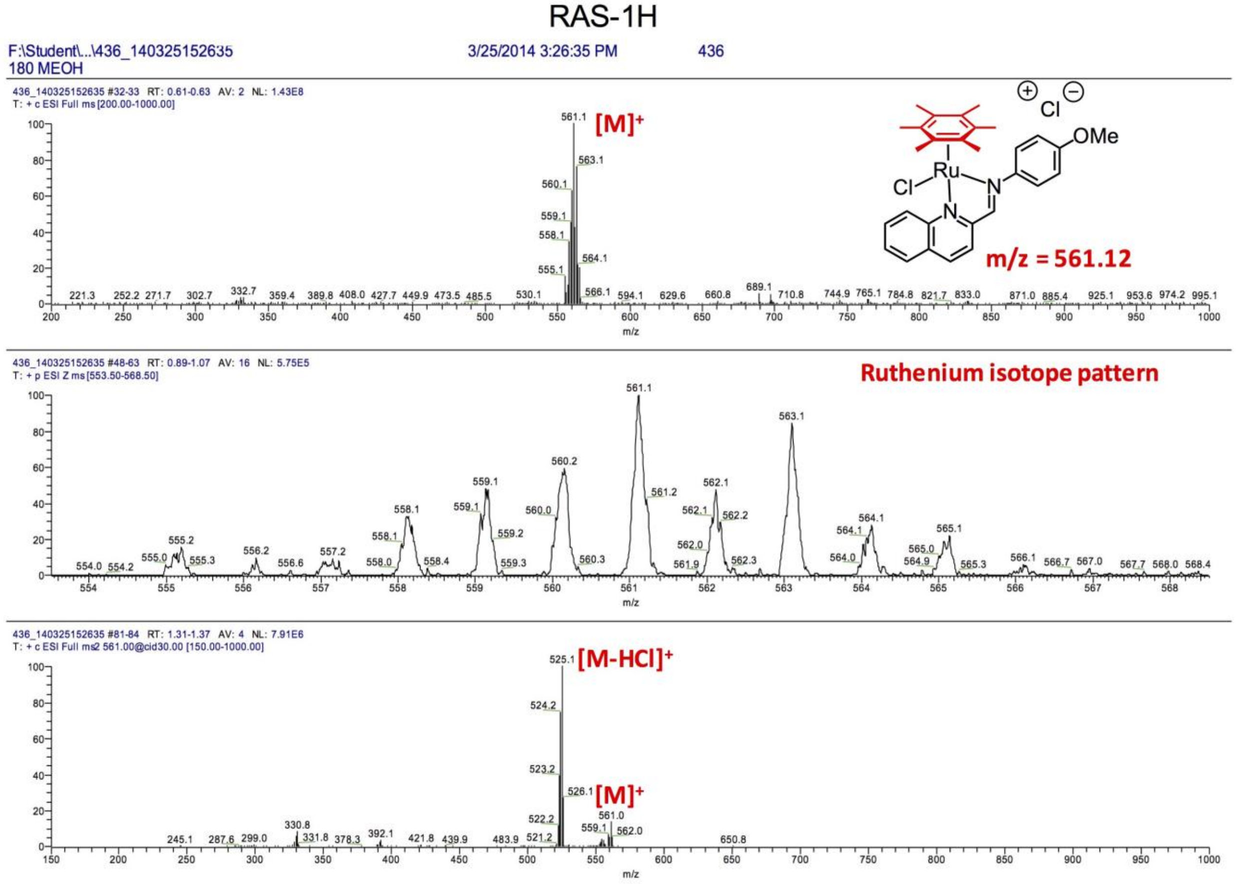

Figure S2. ESI-MS spectrum of RAS-1H. (Top panel) Full MS spectrum; (Middle Spectrum) Zoom Scan of molecular ion peak. Ruthenium and chloride isotope pattern is observed; (Bottom panel) Tandem MS-MS spectrum. Expected fragmentation pattern was observed.

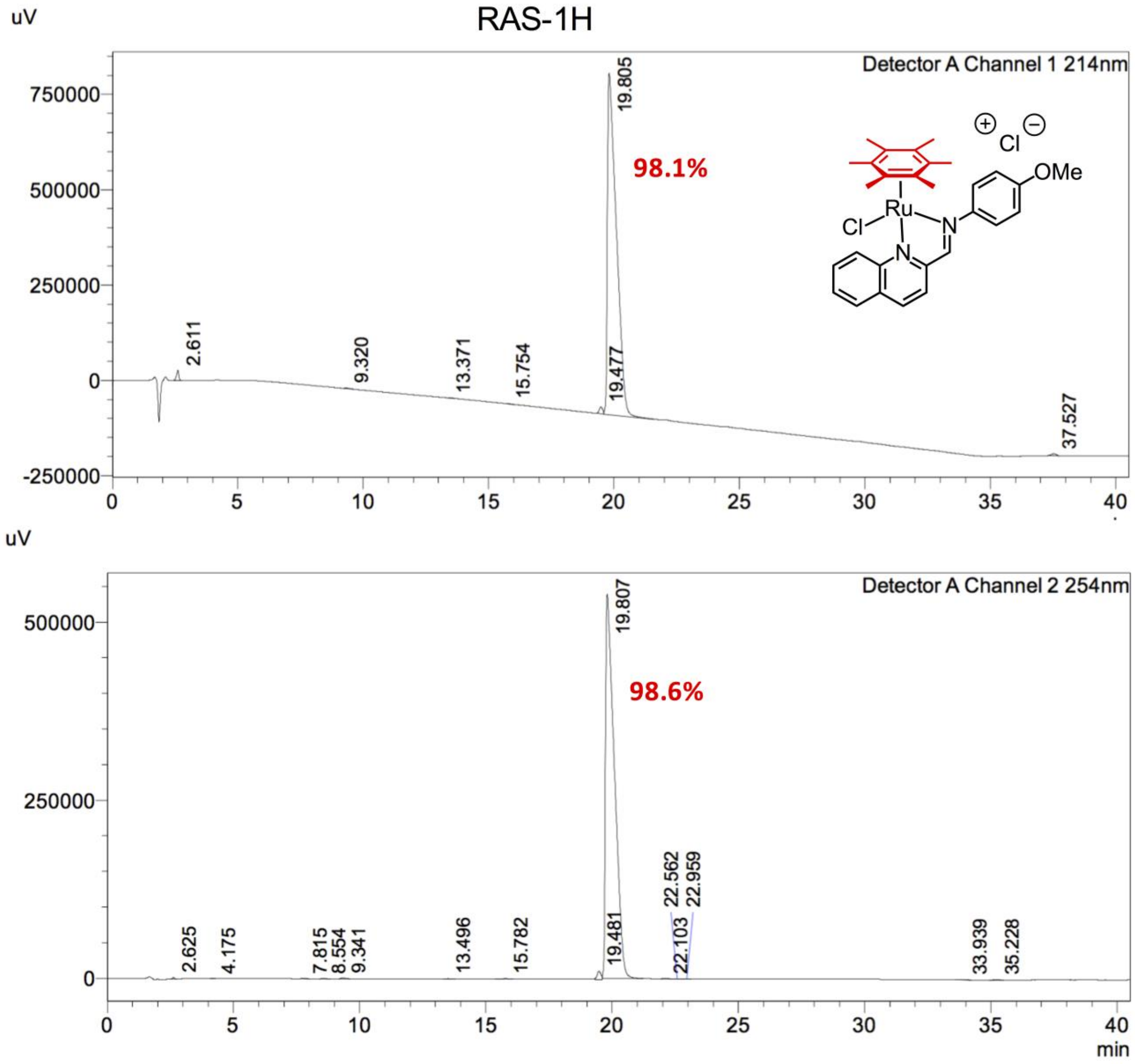

Figure S3. RP-HPLC Chromatograms of RAS-1H. (Top panel) UV detector wavelength at 214 nm. (Bottom panel) UV detector wavelength at 254 nm.

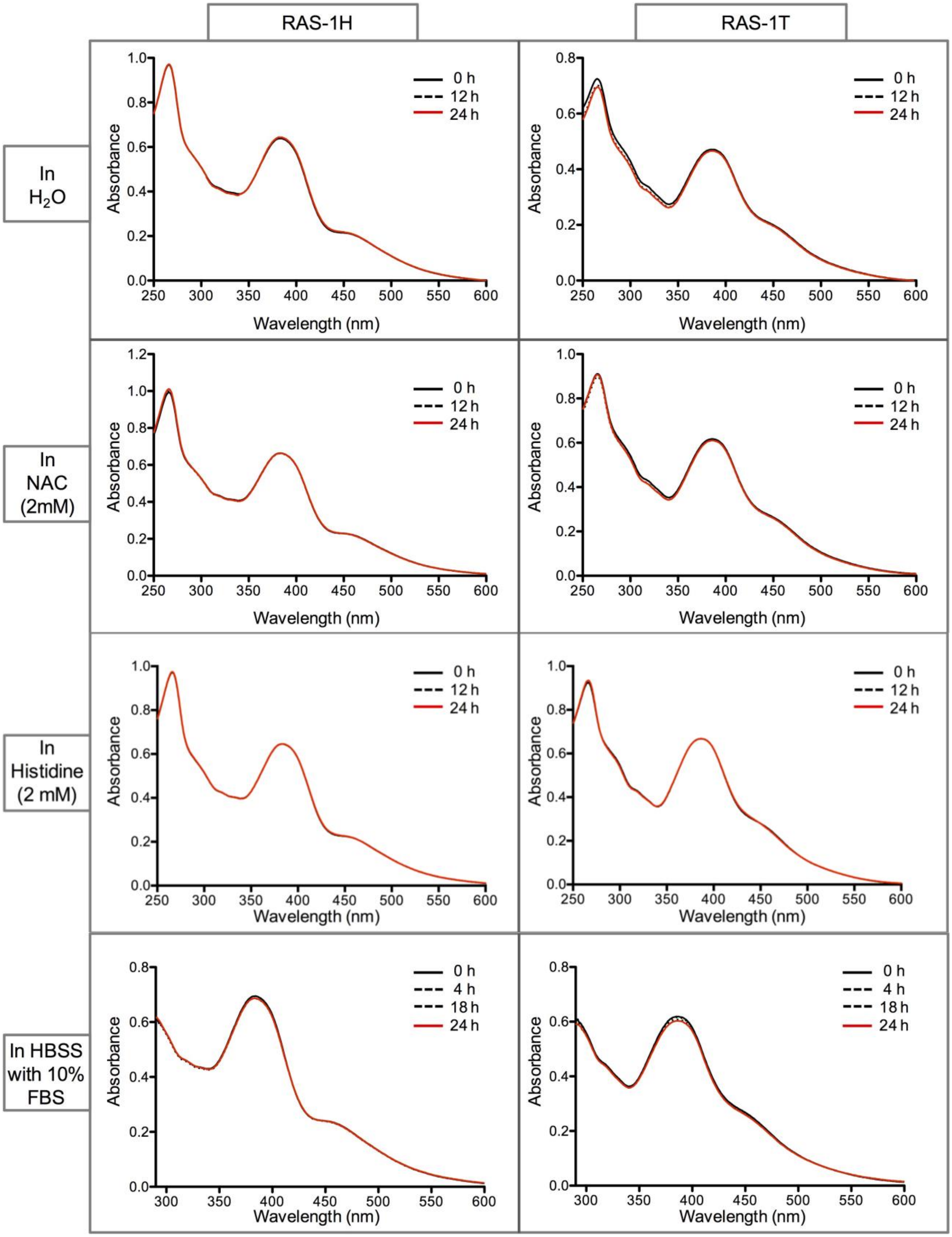

Figure S4. Stability of RAS-1H and RAS-1T in various conditions. Stability of RAS-1H and RAS-1T at 50 µM was determined by observing the change in UV-Vis spectrums over time. In general, the complexes are stable towards hydrolysis and ligand substitution as seen in the lack of change in the spectrums. (HBSS = Hank's Balanced Salt Solution)

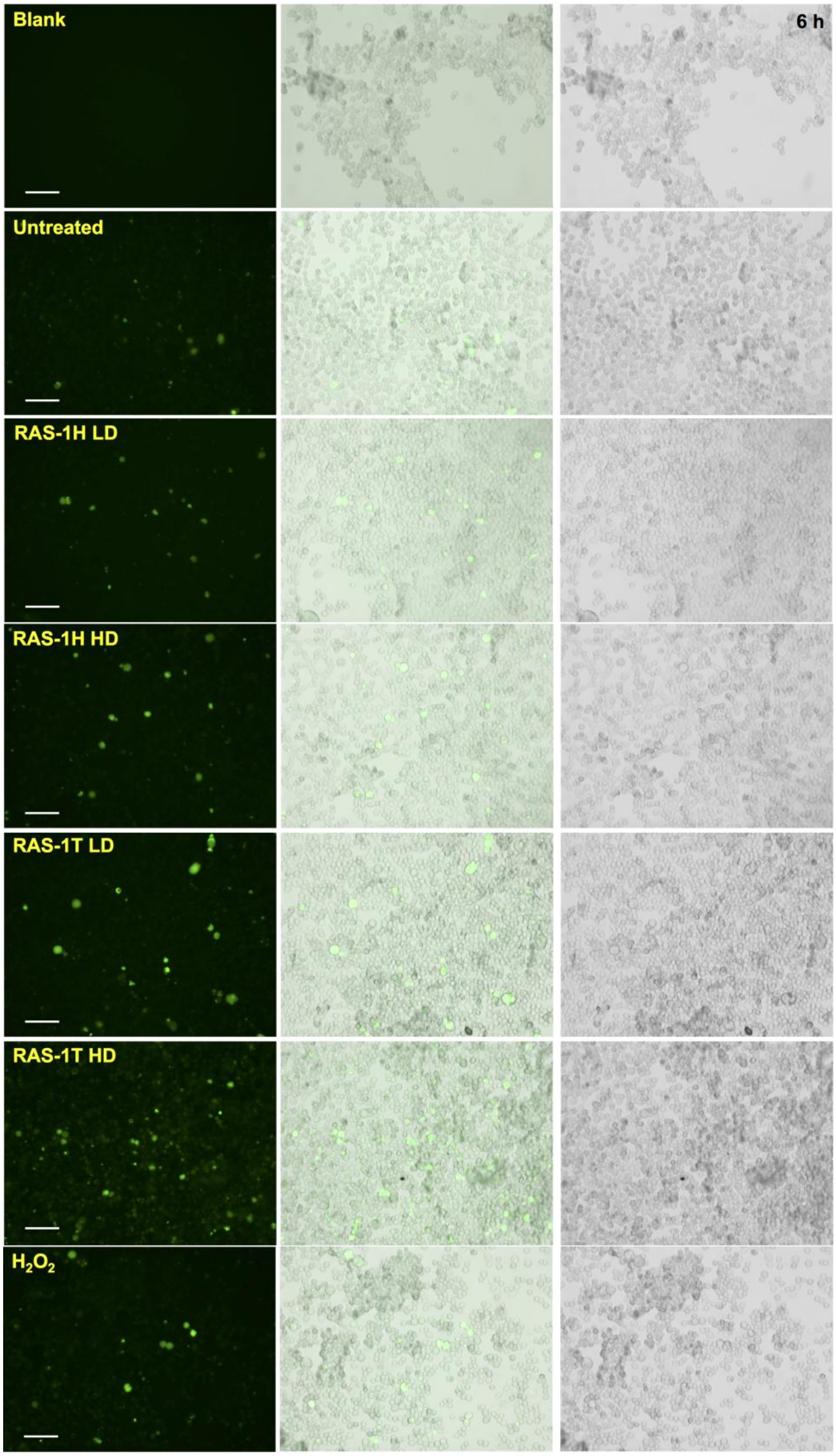

Figure S5a. ROS induction for 6h-treatment. (Left panels) Fluorescence microcopy image taken at 585 nm after incubation with carboxy-H_2DCFDA (20 µM); (Middle panels) overlay; (Right Panels) visible light image (scale bar = 100 µm).

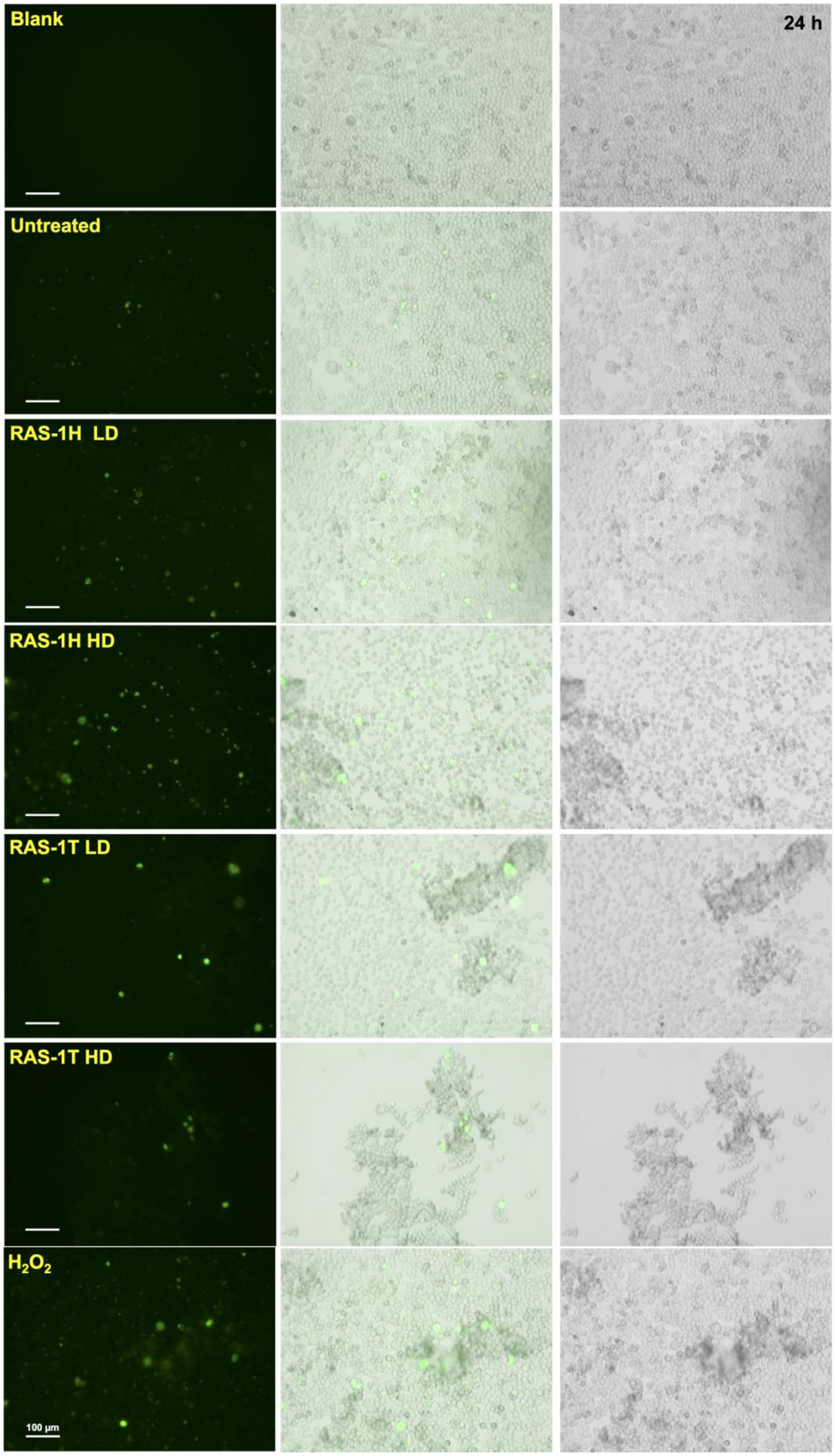

Figure S5b. ROS induction for 24h-treatment. (Left panels) Fluorescence microscopy image taken at 585 nm after incubation with carboxy-H_2DCFDA (20 µM).; (Middle panels) overlay; (Right Panels) visible light image (scale bar = 100 µm).

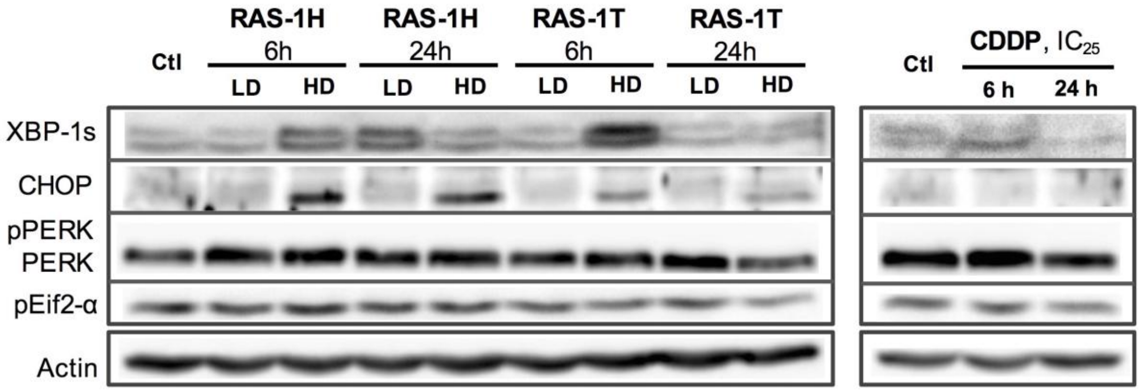

Figure S6. Both RAS-1H and RAS-1T induce ER-stress *via* the IRE1α/XPB-1s pathway. Western blot analysis of proteins markers related to ER-Stress after AGS cells were treated with RAS-1H, RAS-1T and cisplatin for 6h and 24h. Homogeneous protein loading determined with reference to actin.

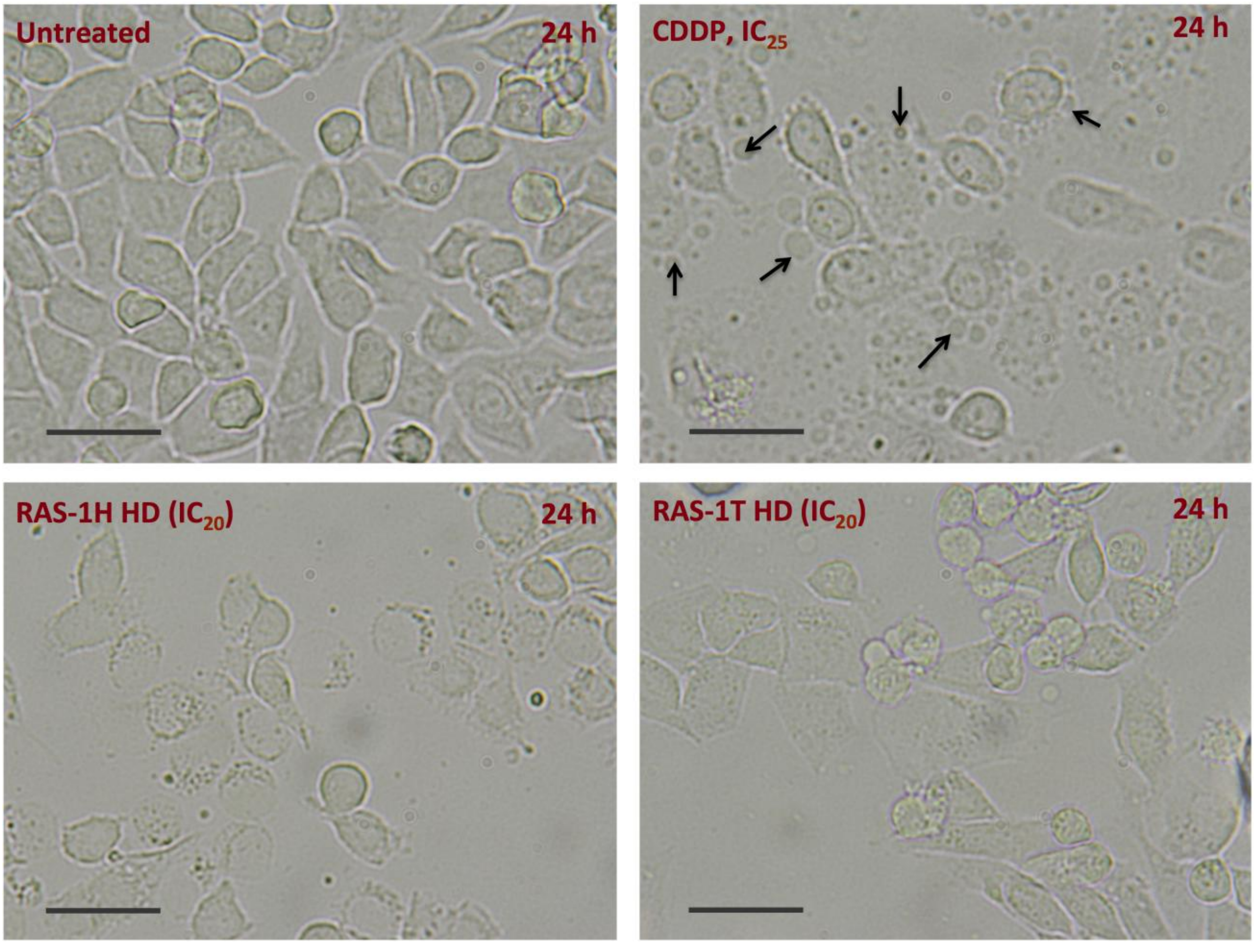

Figure S7. RAS-1H and RAS-1T induce different cell death morphology compared to apoptotic cisplatin. Microscope images of AGS cells treated with RAS-1H, RAS-1T and Cisplatin (scale bar = 100 µm)**.** Examples of smaller apoptotic bodies due to 'budding' are pointed out with black arrows.

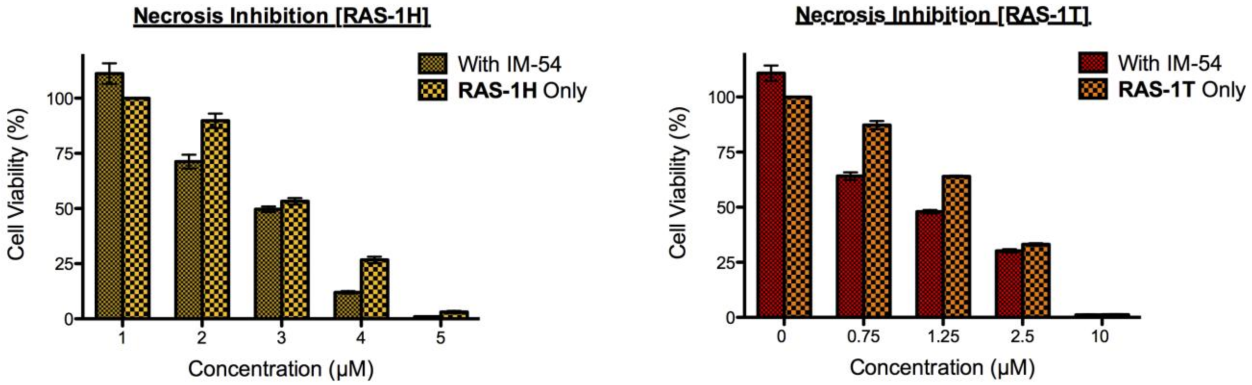

Figure S8. Inhibition of Cell Death MTT Assay. The type of cell death induced by RAS-1H (**1**) and RAS-1T (**2**) was investigated by performing a 48 h cell viability assay in the absence and presence of Necrosis inhibitor, IM-54 (10 μM).

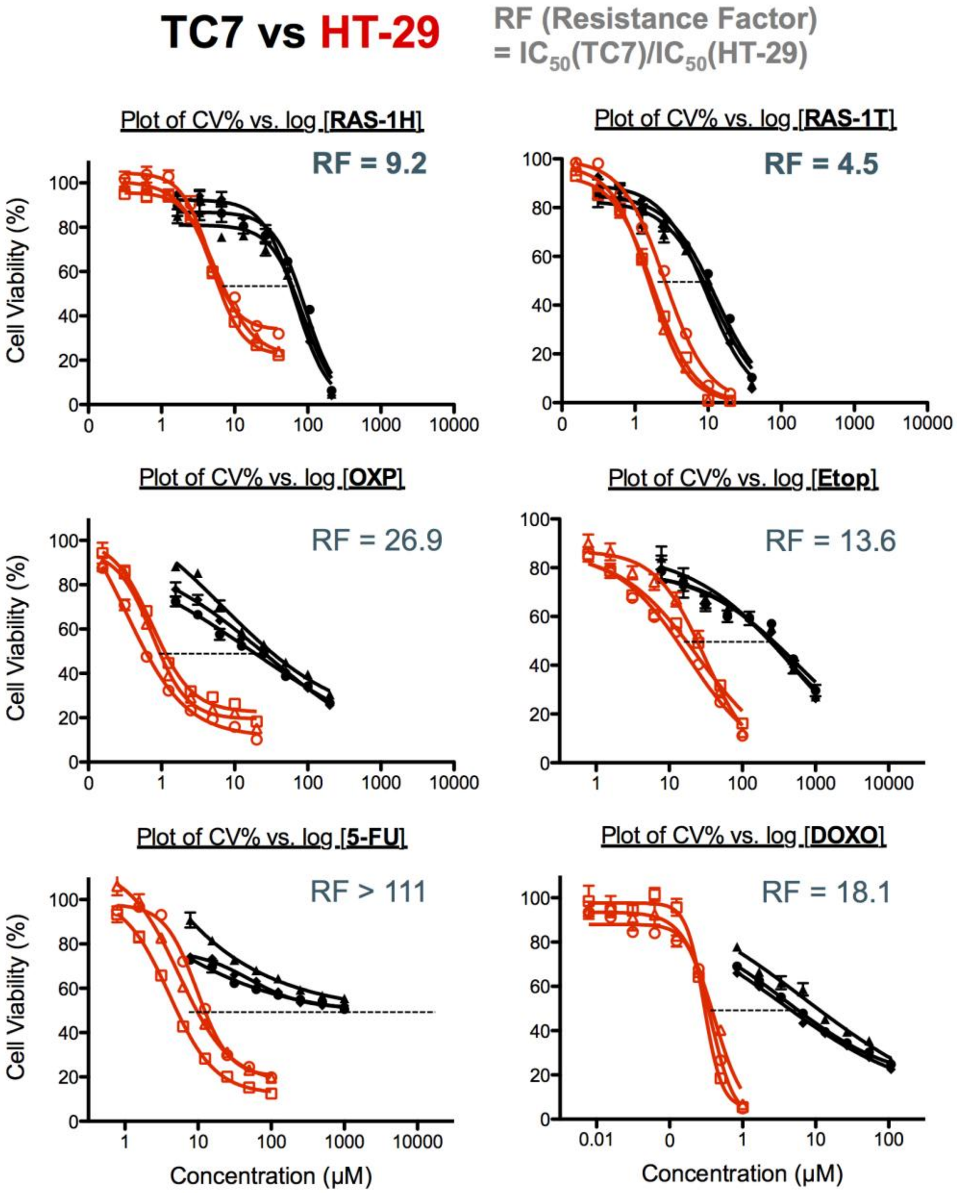

Figure S9a. Comparison of cell viability curves from three independent experiments (TC7 vs HT29).

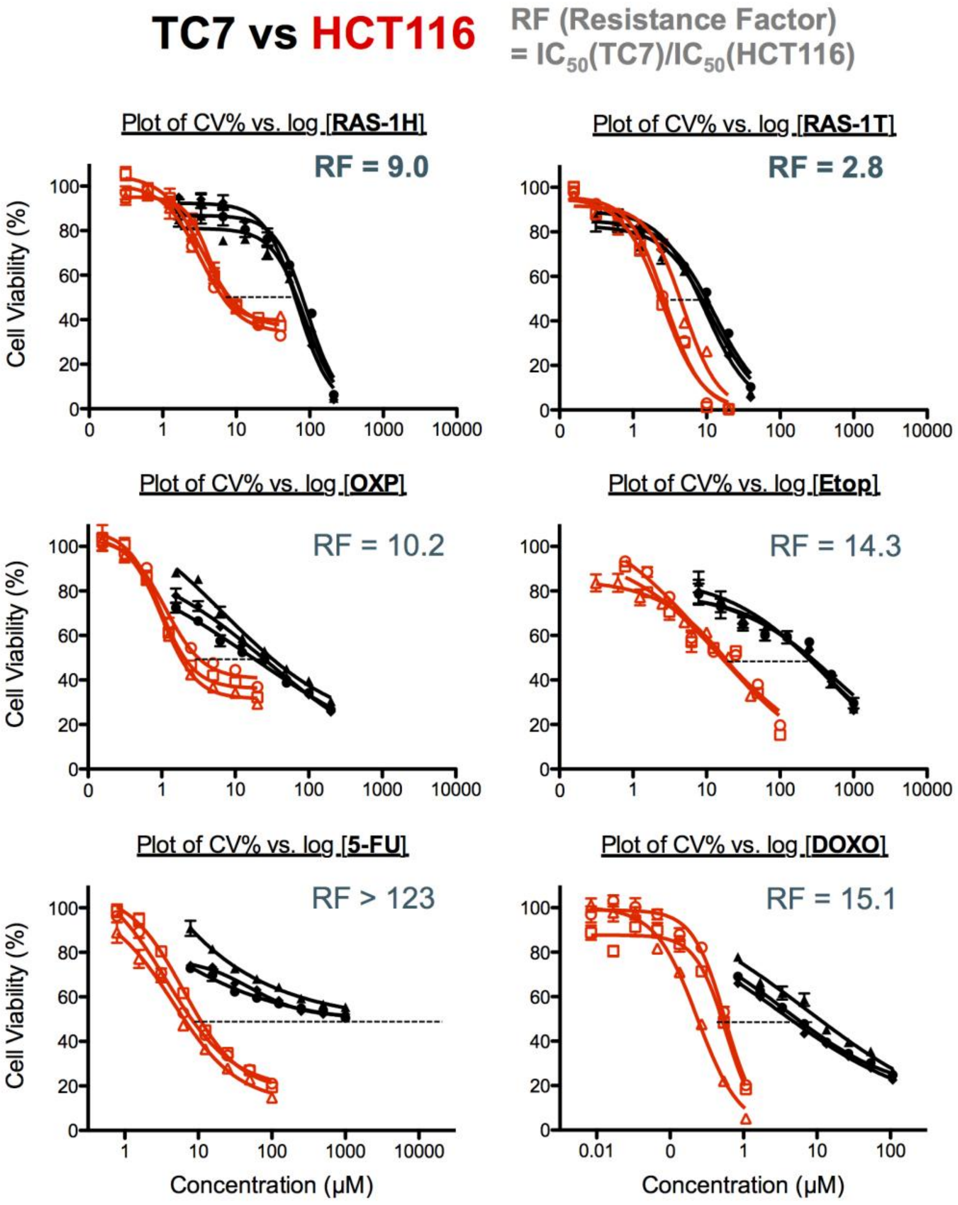

Figure S9b. Comparison of cell viability curves from three independent experiments (TC7 vs HCT116).

SCHEMES

Scheme S1. Simplified antioxidant defense pathway.

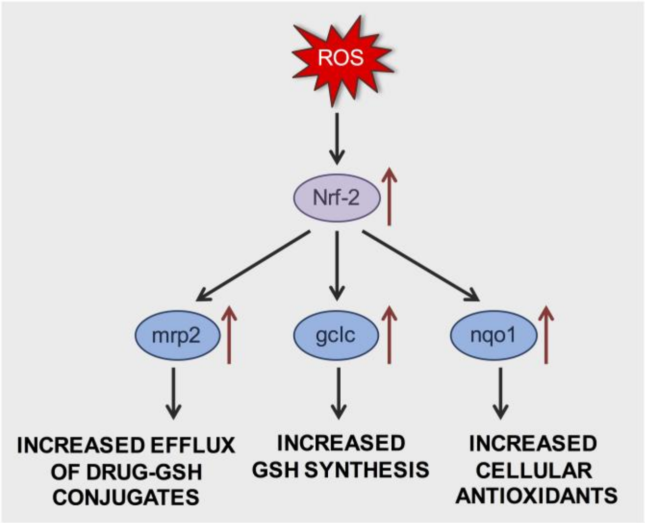

Scheme S2. Simplified ER stress pathway. Black arrows indicated specific pathways induced by RAS complexes.

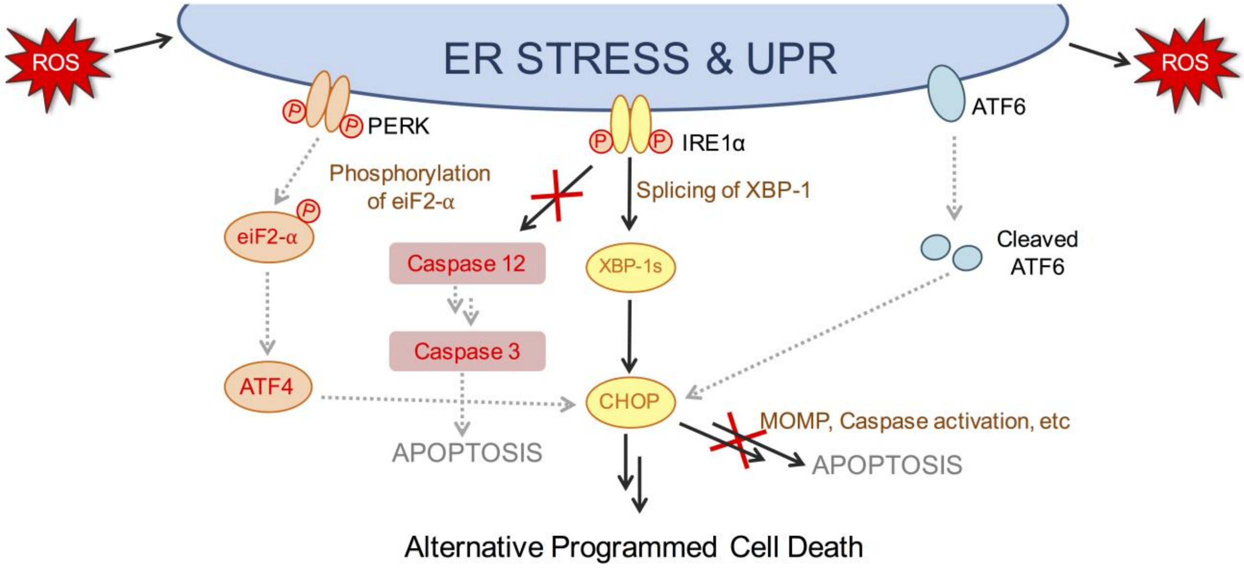

Scheme S3. Contrasting pathway activation between cisplatin and RAS complexes.

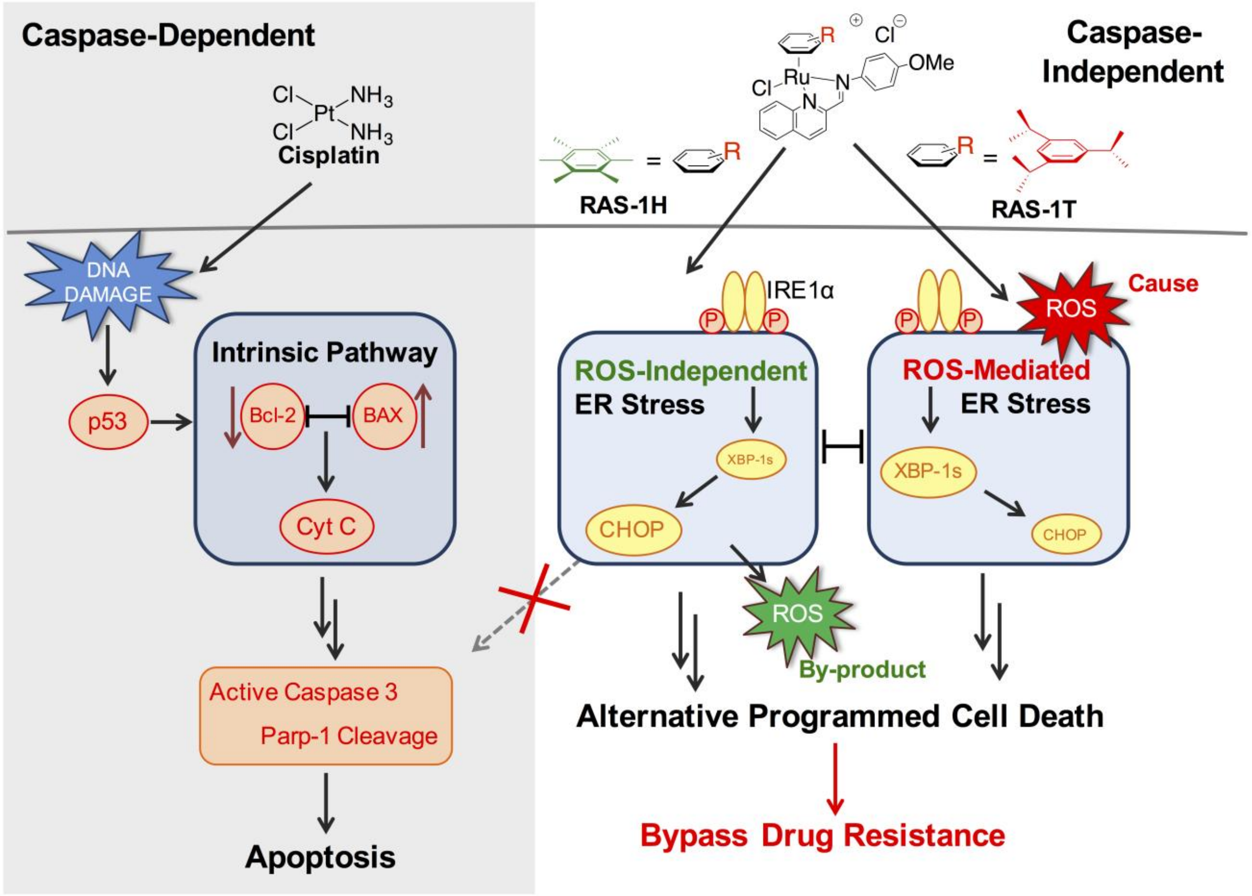

TABLES

Table S1. Log P_{OW} and cytotoxicity data for compound RAS-1H and RAS-1T.

Complex	Log P_{ow}[a]	IC_{50} [μM] [b]			
		HCT116	SW480	AGS	KATOIII
**RAS-1H**	-1.40 ± 0.15	5.76 ± 1.22	34.7 ± 19.3	3.04 ± 0.91	13.7 ± 5.0
**RAS-1T**	-0.85 ± 0.02	1.19 ± 0.12	4.07 ± 1.35	1.01 ± 0.07	1.22 ± 0.24
**Oxaliplatin**	n.d.	1.22 ± 0.18	1.08 ± 0.36	n.d.	n.d.
**Cisplatin**	n.d.	n.d.	n.d.	34.7 ± 0.8	8.64 ± 0.11

[a]Log P_{ow} values determined via the shake-flake method against 1:1 *n*-octanol:H_2O partitioning. [b]IC_{50} values is the concentration of Ru complexes required to inhibit 50% of cell growth with respect to control groups, measured by MTT assay after 48 h of incubation. Data obtained are based on the average of three independent experiments, and the reported errors are the corresponding standard deviations. The IC_{50} were corrected using actual [Ru] determined using ICP-OES.

Table S2: Cytotoxicity data for drug-resistant and drug-sensitive colorectal cell lines.

Complex	[IC_{50}] /μM[a]			Resistance Factor	Resistance Factor
	TC7	HT-29	HCT116	$[IC_{50}]_{TC7}/[IC_{50}]_{HT29}$	$[IC_{50}]_{TC7}/[IC_{50}]_{HCT116}$
**RAS-1H**	65.8 ± 7.3	7.15 ± 0.66	7.33 ± 0.78	9.2	9.0
**RAS-1T**	8.74 ± 0.94	1.93 ± 0.56	3.08 ± 1.04	4.5	2.8
**Oxaliplatin**	23.4 ± 6.6	0.87 ± 0.25	2.29 ± 0.7	26.9	10.2
**Etoposide**	238 ± 16	17.5 ± 5.5	16.6 ± 0.8	13.6	14.3
**5-Fluorouracil**	> 1000	8.99 ± 3.62	8.16 ± 1.97	>111	>123
**Doxorubicin**	6.51 ± 3.27	0.36 ± 0.04	0.43 ± 0.17	18.1	15.1

[a]IC_{50} values is the concentration of Ru complexes required to inhibit 50% of cell growth with respect to control groups, measured by MTT assay after 48 h of incubation. Data obtained are based on the average of three independent experiments, and the reported errors are the corresponding standard deviations. The IC_{50} were corrected using actual [Ru] determined using ICP-OES.

REFERENCES

[1] M. J. Chow, C. Licona, D. Y. Q. Wong, G. Pastorin, C. Gaiddon, W. H. Ang, *J. Med. Chem.* **2014**, *57*, 6043-6059.

[2] I. Chantret, A. Rodolosse, A. Barbat, E. Dussaulx, E. Brot-Laroche, A. Zweibaum, M. Rousset, *J. Cell Sci.* **1994**, *107*, 213-225.
